# A-Type Lamins Maintain the Positional Stability of DNA Damage Repair Foci in Mammalian Nuclei

**DOI:** 10.1371/journal.pone.0061893

**Published:** 2013-05-02

**Authors:** Robert Mahen, Hiroyoshi Hattori, Miyoung Lee, Pooja Sharma, Anand D. Jeyasekharan, Ashok R. Venkitaraman

**Affiliations:** The Medical Research Council Cancer Cell Unit, Hutchison/MRC Research Centre, Cambridge, United Kingdom; Dana-Farber/Harvard Cancer Institute, United States of America

## Abstract

A-type lamins encoded by *LMNA* form a structural fibrillar meshwork within the mammalian nucleus. How this nuclear organization may influence the execution of biological processes involving DNA transactions remains unclear. Here, we characterize changes in the dynamics and biochemical interactions of lamin A/C after DNA damage. We find that DNA breakage reduces the mobility of nucleoplasmic GFP-lamin A throughout the nucleus as measured by dynamic fluorescence imaging and spectroscopy in living cells, suggestive of incorporation into stable macromolecular complexes, but does not induce the focal accumulation of GFP-lamin A at damage sites. Using a proximity ligation assay and biochemical analyses, we show that lamin A engages chromatin via histone H2AX and its phosphorylated form (γH2AX) induced by DNA damage, and that these interactions are enhanced after DNA damage. Finally, we use three-dimensional time-lapse imaging to show that *LMNA* inactivation significantly reduces the positional stability of DNA repair foci in living cells. This defect is partially rescued by the stable expression of GFP-lamin A. Thus collectively, our findings suggest that the dynamic structural meshwork formed by A-type lamins anchors sites of DNA repair in mammalian nuclei, providing fresh insight into the control of DNA transactions by nuclear structural organization.

## Introduction

How the dynamic three-dimensional organization of the mammalian nucleus influences fundamental processes like DNA repair is unclear [1], [2]. We have studied in this context the role of A-type lamins, the intermediate-filament proteins lamin A and C encoded by *LMNA*, which form a structural scaffold at the nuclear membrane and within the nuclear interior [3], [4] thought to bind to chromatin [5]. Mutations in *LMNA* occur in human progeroid syndromes including Hutchinson Gilford Progeria Syndrome [6]. Cells from patients with Hutchinson-Gilford Progeria Syndrome [7] or from mouse or cell culture models of the disease [8], [9] have both increased levels of DNA damage, and defects in DNA repair foci assembly formation. Similarly, *LMNA* inactivated cells have increased levels of the DNA damage marker γH2AX, chromatid breaks and increased sensitivity to ionising radiation (IR) [10], [11]. Although these observations implicate lamin A/C in cellular responses to DNA damage, the mechanism for their participation remains unclear. One possible role is suggested by recent studies in several model systems suggesting that the movement of damaged chromatin is actively regulated. Thus, movement may be restrained to avoid potentially deleterious chromosome translocations [12], or promoted, to position DNA damage sites within favourable chromatin environments for repair [13]–[16] and possibly promote homologous chromosome pairing and DNA repair pathway choice [17], [18]. These observations prompted us to characterize changes in the dynamics and biochemical interactions of lamin A/C following DNA damage. Here, we suggest a previously unrecognized function for A-type lamins in maintaining the positional stability of DNA repair foci in mammalian nuclei.

## Results and Discussion

### GFP-lamin A does not accumulate at sites of DNA damage

**Figure 1:**
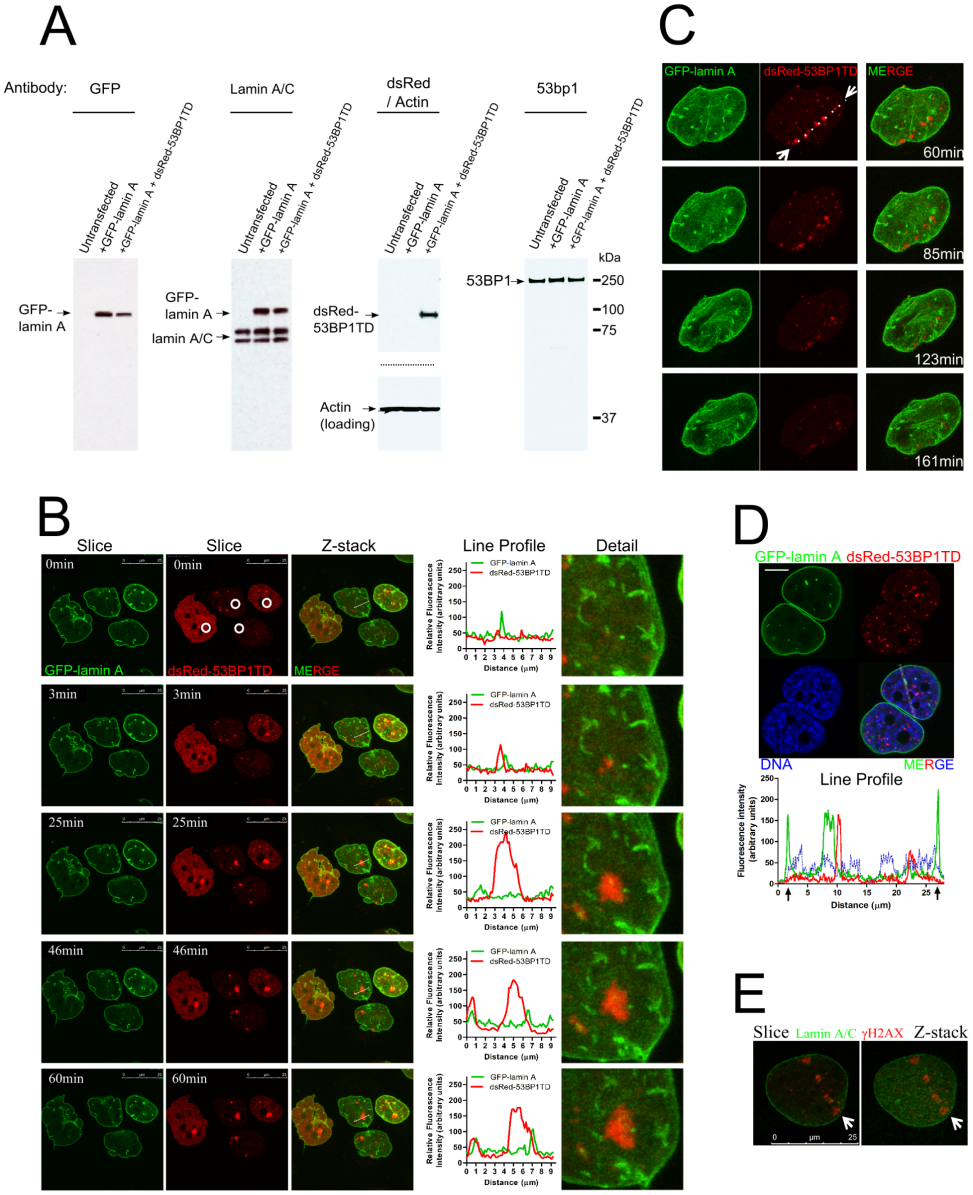
GFP-lamin A does not accumulate at sites of DNA damage. (**A**) Western blot analysis of U2OS cells stably co-expressing GFP-lamin A and dsRed-53BP1TD, using multiple different antibodies as indicated. The whole gel is shown to indicate the absence of erroneously truncated forms of the proteins which might complicate analyses, and horizontal dashed lines indicate where the membrane was cut to incubate with different antibodies. (**B**) DNA damage was induced focally using a 405 nm laser following Hoechst 43332 pre-sensitisation, in one spot per cell (denoted by the white circles). GFP-lamin A and dsRed-53BP1TD were followed simultaneously by time-lapse imaging at one minute intervals. The images show example timepoints and the line profile graphs quantitate the fluorescence intensity of both channels in the zoomed region entitled “detail”. (**C**) DNA damage was induced focally using a 365 nm micropoint laser (Andor) and GFP-lamin A dsRed-53BP1TD were followed with time-lapse imaging using one minute intervals. The images are maximum intensity projections from confocal z-stacks, showing timepoints 60–161 min after DNA damage induction. The white arrows and white dashed line denote the route taken by the 365 nm laser used to induce DNA damage prior to time-lapse imaging. (**D**) Representative confocal image and line intensity profile of a fixed GFP-lamin A/dsRed-53BP1TD cell stained for DNA with Hoechst 33342 after treatment with the radio-mimetic topoisomerase inhibitor etoposide (5 µM). The black arrows on the x-axis of the line intensity profile graph indicate the position of the lamina, and the white dashed opaque line on the image indicates the position of the line profile shown in the graph. Scale bar 10 µm. (**E**) Confocal images of a cell damaged focally with a micropoint 365 nm laser (denoted by white arrows) and stained for the marker of DNA damage γH2AX and endogenous lamin A/C.

We simultaneously followed in living cells the localization of both lamin A and the DNA repair factor 53BP1, using time-lapse imaging with high temporal and spatial resolution, in a clonal U2OS cell line stably co-expressing GFP-lamin A and dsRed fused to the mTudor domain of 53BP1 (dsRed-53BP1TD). Each fusion protein was detected in western blots as a single species with the expected molecular mass using several different antibodies (**Fig. 1A**). dsRed-53BP1TD formed visible foci in unchallenged cells as previously reported [19], which increased in number after the treatment of cells with IR, and were confirmed as sites of DNA damage by their co-localization with staining for the serine-139 phosphorylated form of histone H2AX (γH2AX; **Fig. S1**).

dsRed-53BP1TD accumulated in foci at sites of DNA damage induced within the nuclei of living cells either with a 405 nm laser after pre-sensitization with Hoechst 33342, or with a pulsed 365 nm micropoint laser. However, GFP-lamin A did not exhibit such accumulation, either during the formation of DNA repair foci (**Fig. 1B**, **Video S1**, **S2**, **S3**), or at later time-points, during their resolution (**Fig. 1C**, **Video S4**). We obtained similar results when observing endogenously occurring DNA damage foci in asynchronously growing cells, and after different forms of DNA damage induced by IR or the radio-mimetic topoisomerase inhibitor, etoposide (**Fig. 1D**). Furthermore, staining for endogenous lamin A/C with multiple different antibodies also showed no significant accumulation of lamin A/C at sites of repair (**Fig. 1E**).

Intra-nuclear accumulations of GFP-lamin A occurred within the nucleoplasm, consistent with previous reports [20]. However, DNA repair foci did not co-localize with these regions, even when induced to form directly on them (**Fig. 1B, 1C**), in which case they remained adjacent (**Fig. 1B**, line profile and detail). Similarly, dsRed-53BP1TD foci were observed directly adjacent to the large enrichment of GFP-lamin A at the nuclear membrane (the lamina), but there was no significant co-localization between the two structures, or to heterochromatic regions of chromatin marked by intense Hoechst 33342 staining (**Fig. 1D**). Collectively, these observations suggest that lamin A fails to accumulate in, or disperse from, repair foci on damaged chromatin.

### DNA damage induces a decrease in GFP-lamin A mobility throughout the nucleus

**Figure 2:**
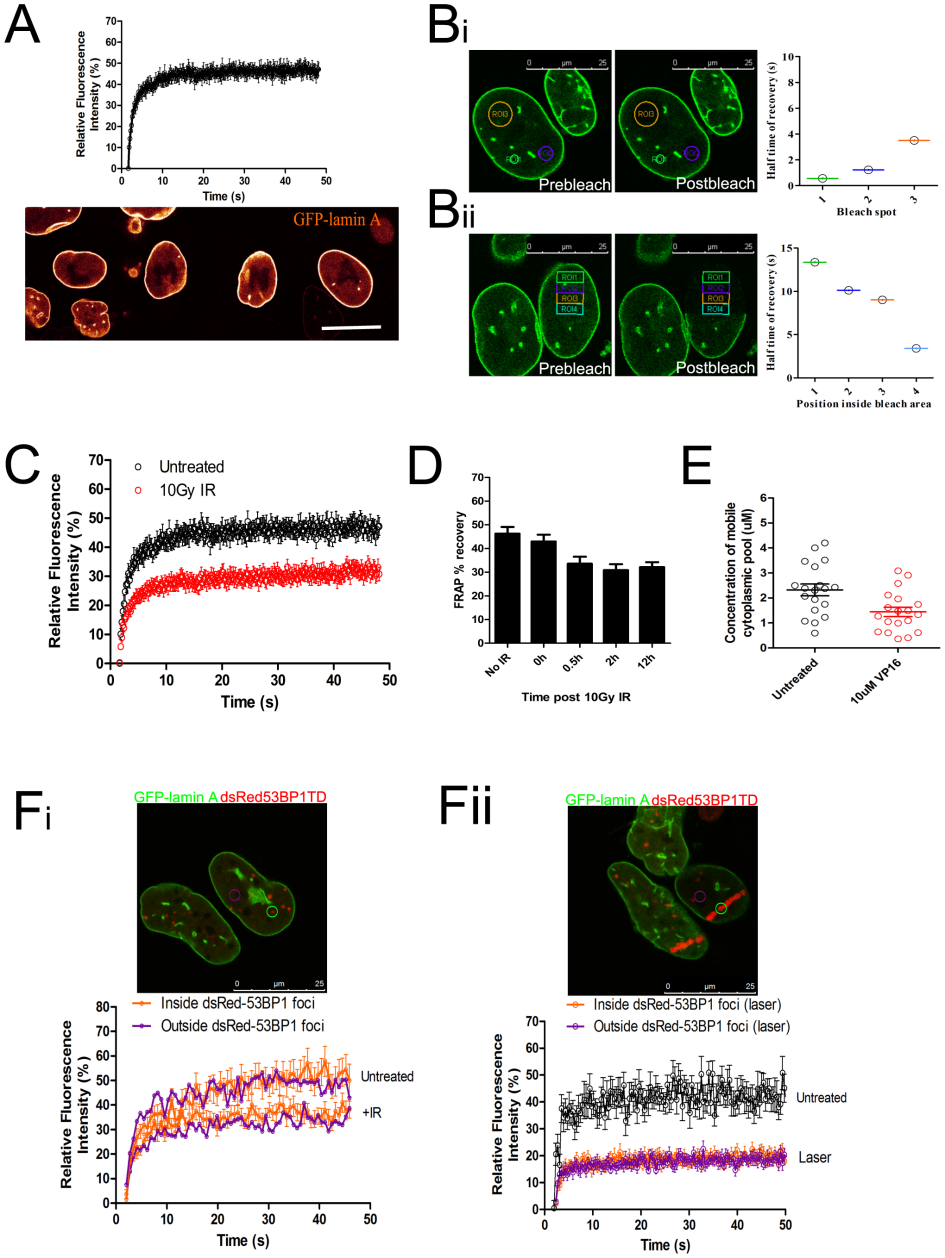
DNA damage induces a pan-nuclear decrease in GFP-lamin A mobility. (**A**) FRAP data from the nucleoplasm of U2OS cells stably expressing GFP-lamin A. GFP-lamin A was bleached in a circle ∼1 µm in diameter using 100% laser power and fluorescence recovery was monitored using 3% laser power. The image shows four GFP-lamin A cells bleached three minutes previously with the shapes of the letters “LMNA”, demonstrating relatively little movement of the protein on this timescale. Scale bar 20 µm. (**B**) Shows the results of FRAP experiments on GFP-lamin A. In (**i**) three different bleach spot sizes were used, denoted ROI1, ROI2, ROI3. The rate of recovery into each bleach spot was monitored and plotted as the halftime of recovery, shown on the graph and calculated as described in **methods**. In (**ii**) half of the cell was bleached, and recovery was monitored at varying distances from the non-bleached area, denoted ROI1, ROI2, ROI3 and ROI4. (**C**) The graph plots the average fluorescence recovery curves (+/− s.e.m.) taken from 14 cells as described in (**A**), either before (black) or after (red) DNA damage induction using 10Gy IR. (**D**) GFP-lamin A FRAP recovery curves were taken at varying times after 10Gy IR. The bar graph shows the mean % recovery (+/− s.e.m) of GFP-lamin A FRAP recovery curves. (**E**) Fluorescence correlation spectroscopy measurements were taken in GFP-lamin A cells either untreated or after incubation with the DNA damaging drug etoposide. Each dot shows the average concentration of mobile GFP-lamin A in the cytoplasm of a single cell during a 45 second measurement, calculated from a fit of the G0 of the autocorrelation function as described in **methods**. (**F**) GFP-lamin A FRAP measurements were taken inside and outside regions of DNA damage marked by dsRed-53BP1TD, either in asynchronously growing cells or in cells damaged using a 365 nm laser as described in **Fig. 1C**. The images show example cells in which measurements were taken and the graphs show mean recovery curves +/− s.e.m taken from 15 cells in two independent experiments.

We used fluorescence recovery after photobleaching (FRAP) to study the dynamic reaction-diffusion behaviour of GFP-lamin A in living cells after DNA damage. Consistent with previous work [21]–[23] GFP-lamin A in the nucleoplasm of undamaged cells exhibited a low overall recovery in FRAP measurements of its dynamic exchange over a period of ∼1 min (**Fig. 2A**), suggesting that the majority of GFP-lamin A in the nuclear interior participates in stable, long-lived binding interactions that retard its mobility on this time-scale. However, ∼40% of nucleoplasmic GFP-lamin A exhibits dynamic recovery in FRAP, and so we examined the relative contributions of diffusion and binding to the mobility of this dynamic fraction using previously described analyses [24], [25]. Thus, increasing the size of the GFP-lamin A bleach spot (**Fig. 2B** **i**), or its distance from a fluorescent area (**Fig. 2** **Bii**), increased the T^1/2^ of the fluorescence recovery curves. This suggests that there is a dynamically exchanging steady-state population of nucleoplasmic GFP-lamin A whose mobility is retarded by more rapid binding interactions, since its recovery time varies depending on the time taken to diffuse.

Interestingly, IR induced DNA damage significantly decreased the proportion of the dynamic pool of GFP-lamin A (**Fig. 2C**) from 46+/−3% to 31+/−3% (p = 0.003, t-test), consistent with recruitment into stable, long-lived binding interactions. This effect was apparent within 30 min after exposure to 10Gy IR, and persisted for >2 h (**Fig. 2** **D**). It was also confirmed by analyses using fluorescence correlation spectroscopy, visible as a decrease in mobile cytoplasmic GFP-lamin A concentration diffusing through the confocal volume (**Fig. 2E**). Our findings suggest that the effect arises from nucleus-wide changes in the mobility of GFP-lamin A. Thus, nucleoplasmic GFP-lamin A mobility was decreased within or outside sites of DNA damage marked by dsRed-53BP1TD foci, whether measured in asynchronously dividing cells, in IR-treated cells, or in cells with focal DNA damage induced by a 365 nm high-intensity laser (**Fig. 2F**). Together, these observations (**Fig. 2**) confirm that much (∼60%) of nucleoplasmic GFP-lamin A is incorporated in long-lived interactions that reduce its mobility [21], [23], but reveal that the remainder of the protein is dynamic [26] in undamaged nuclei, and unexpectedly recruited to an immobile pool within 30 min after DNA damage.

### Altered lamin A–H2AX interaction after DNA damage

**Figure 3:**
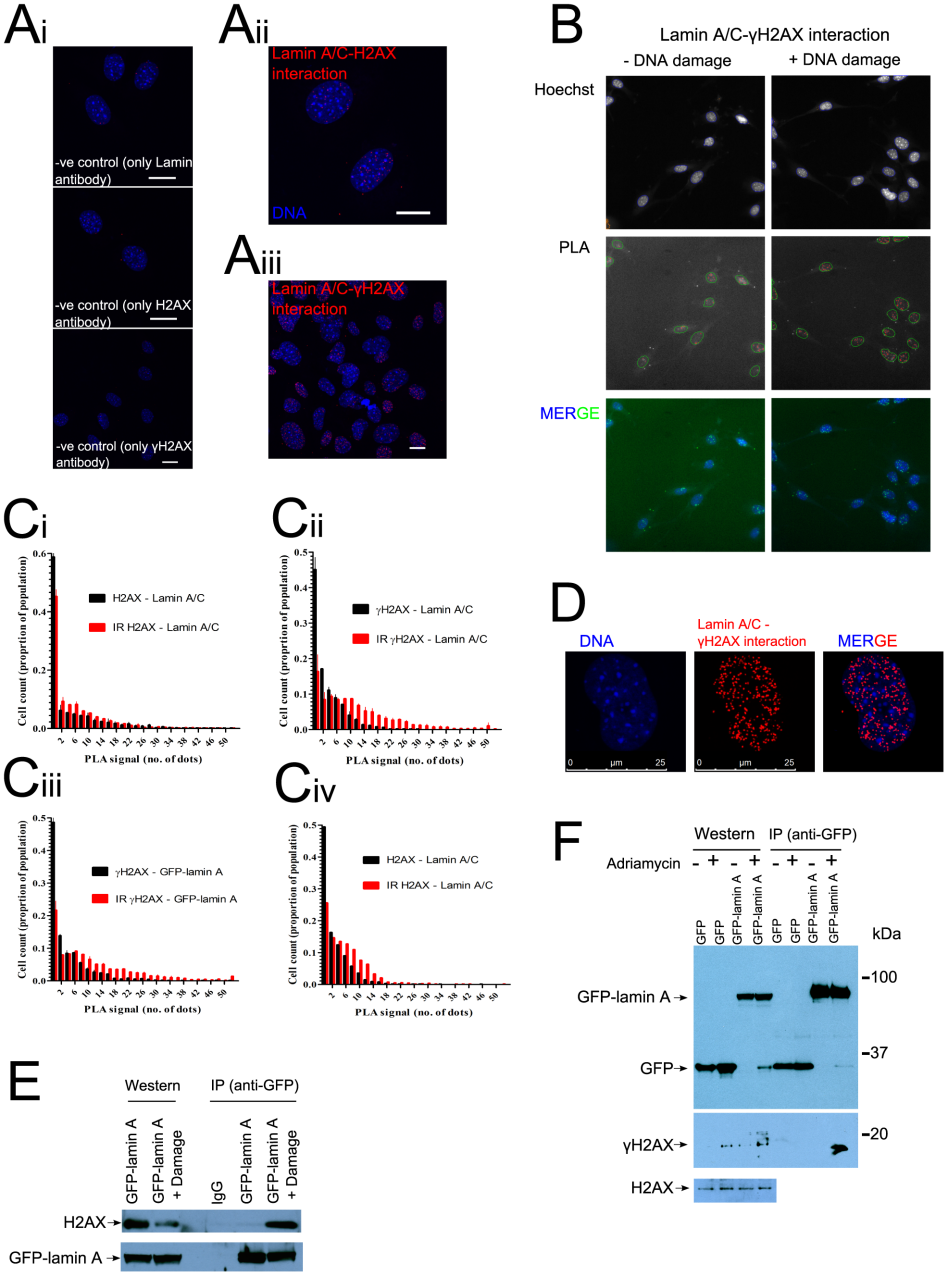
Altered lamin A/C-H2AX interaction after DNA damage. (**A**) Example immunofluorescent images of lamin-Histone H2AX interactions measured by proximity ligation assay in murine embryonic fibroblasts. Red dots represent the PLA signal, produced from antibodies in close proximity (<40 nm), and blue shows DNA stained by Hoechst 33342 dye. (**i**) Negative controls containing an antibody against only one interaction partner (either lamin A/C, H2AX or γH2AX). (**ii**) Sample containing both lamin A/C and H2AX antibodies (**iii**) Sample containing both lamin A/C and γH2AX antibodies. Scale bars 20 µm. (**B**) Automated microscopy was used to automatically quantitate PLA signal −/+ DNA damage in LMNA−/− GFP-lamin A cells stained with Hoechst 33342. The images show one example field of cells each for − and + DNA damage, in two different fluorescent channels containing Hoechst 33342 staining (top) and PLA signal (middle). Images are overlayed with automated identification of: cells as objects (blue), cell nuclei (green) and PLA signal (red dots). The bottom panel shows a colour overlay of PLA (green dots) and Hoescht staining (blue). (**C**) The histograms show the number of PLA dots +/− s.e.m in >2000 cells from two independent experiments. (**i**–**iii**) show results from murine embryonic fibroblasts and (**iv**) is from human cells. (**D**) The image shows the location of the PLA signal obtained from interaction between lamin A/C and γH2AX relative to chromocenters demarked by Hoechst 33342 staining in one single nucleus. (**E**) GFP-lamin A - H2AX complexes were found by co-immuno-pulldown. LMNA−/− GFP-lamin A cells were lysed in NP40 buffer and GFP-lamin A was isolated using protein A/G beads and anti-GFP antibody. The eluted complexes were probed by western blot with antibodies against H2AX, IgG and lamin A/C. (**F**) GFP-lamin A - γH2AX complexes were found by co-immuno-pulldown. 293T cells, transfected with either GFP-lamin A or GFP alone, were processed as described in (**E**) and the eluted samples were probed by western blot with antibodies specific to GFP and γH2AX.

We next examined the interactions of A-type lamins with the variant histone H2AX, the phosphorylated form (γH2AX) of which accumulates rapidly at sites of DNA breakage [27]. We used the *in situ* proximity ligation assay (PLA) to detect direct *in vivo* protein-protein interactions between endogenous proteins based on very close proximity (<40 nm) between secondary antibodies directed against them [28]. Short DNA oligonucleotides which are attached to these secondary antibodies serve as a template for rolling circle DNA synthesis, resulting in a PLA signal in the form of a spot. Positive control measurements showed that the PLA signal was proportional to the number of interactions, with relatively low levels of background signal in negative controls in our experimental setup (**Fig. S2,** **Fig. 3A**). The PLA signal in images was quantitated automatically using a high-content microscope (**Fig. 3B** **;** Cellomics Arrayscan), ensuring both an unbiased analysis and large sample size (>1000 cells). Lamin A/C-H2AX interactions were low in undamaged nuclei and became more apparent after exposure to IR (**Fig. 3C** **i**). The interaction of A-type lamins with phosphorylated γH2AX increased significantly (p = 0.004, Mann-Witney U test) after DNA damage (**Fig. 3C** **ii)**. Measurements were normalised to controls using single antibody incubations alone (**Methods**) to exclude a rise in non-specific background signal from γH2AX staining in damaged nuclei. Moreover, in A-type lamin-deficient *LMNA*−/− cells [29], [30] stably expressing GFP-lamin A (*LMNA*−/− GFP-lamin A; **Fig. S3**) a similar PLA signal was detected as in *LMNA*+/+ matched control cells (**Fig. 3C** **iii**), confirming that lamin A rather than lamin C participates in the interaction with H2AX. Increased interactions between endogenous lamin A and H2AX were also evident in human U2OS cells (**Fig. 3C** **iv**). Type-A lamin-H2AX interactions were detected almost exclusively inside the nucleus, and generally occurred outside of chromocenters (**Fig. 3D**), consistent with previous data showing that heterochromatin is refractory to γH2AX formation [31], and that γH2AX formation spans megabases around DNA damage sites [27]. Specific GFP-lamin A-H2AX and GFP-lamin A-γH2AX interactions were also detected by co-immuno-precipitation from cell lysates and indeed, a significantly increased amount of both H2AX and γH2AX could be detected in complex with GFP-lamin A after DNA damage with this independent method (**Fig. 3E, 3F**). Thus, collectively, these results suggest that chromatin is anchored to the nuclear meshwork formed by A-type lamins, consistent with previous work [5], [32], [33], and that these anchorages are altered after DNA damage. The decreased mobile fraction of GFP-lamin A after DNA damage (**Fig. 2**), and its direct interactions with H2AX and γH2AX (**Fig. 3**) are not only consistent with this idea, but also suggest that A-type lamins might anchor DNA repair foci to the nuclear lamin meshwork.

### A-type lamins are required for the positional stability of DNA repair foci

**Figure 4:**
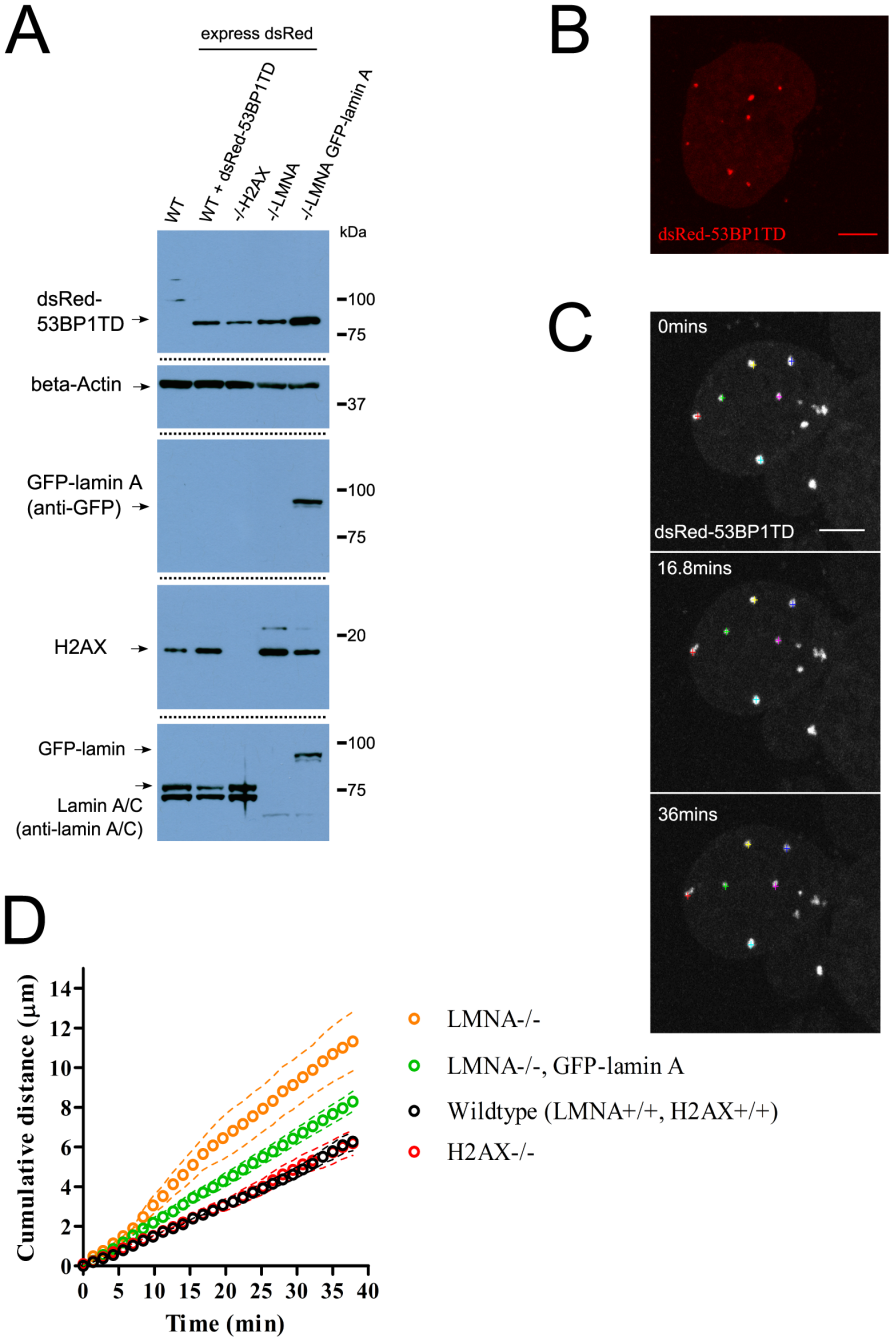
LMNA is required for the positional stability of DNA repair foci. (**A**) Western blots showing the expression of lamin A/C, GFP-lamin A, dsRed-53BP1TD and H2AX in a set of matched murine embryonic fibroblasts. The antibody used to probe cell lysates is given in brackets. The dividing dashed lines divide images taken from the same gel and same exposure. (**B**) Example confocal image of a dsRed-53BP1TD expressing cell, scale bar 8 µm. (**C**) Example timepoints of live cell imaging of a single dsRed-53BP1TD LMNA+/+ cell; coloured crosses indicate the positions of six DNA repair foci from which positional information was taken, scale bar 7 µm. Brightness and contrast settings were altered for display purposes. (**D**) The graph shows the average cumulative distance moved by DNA repair foci in a population of cells (n = 12 cells +/− s.e.m), calculated as described in **methods**.

We therefore measured the positional stability of DNA repair foci marked by dsRed-53BP1TD in matched murine cell lines either wildtype (*LMNA*+/+) or *LMNA* inactivated (*LMNA*−/−) (**Fig. 4A, B** and **Fig. S3B**). Cells treated with 2 µM etoposide to induce DNA damage were followed by 3D time-lapse imaging over ∼40 minutes (**Fig. 4C**), to track the position of dsRed-53BP1TD foci using a previously described method which determines the relative positions of different foci within a single cell so as to exclude whole-cell movements [14]. The mobility of DNA repair foci was significantly increased in *LMNA*−/− cells (**Fig. 4D**), and the expression of GFP-lamin A was sufficient to partially rescue this defect (**Fig. 4D**). Notably and consistent with previous data however [12], the movement of DNA repair foci was not altered in cells lacking H2AX, suggesting either that lamin-H2AX interactions are dispensable for this effect, or that alternative compensatory mechanisms, such as interactions with other chromatin proteins, are operational in the *H2AX*−/− background.

In conclusion, our findings demonstrate that the integrity of A-type lamins is essential to maintain the positional stability of DNA repair foci. The biochemical mechanism underlying this function, as well as its potential role in other DNA transactions shown to be influenced by A-type lamins, such as transcription [5], [34] or telomere arrangement [10], [35], warrants further investigation. Our findings suggest a mechanism wherein the dynamic structural meshwork formed by A-type lamins may spatially anchor the genome [36] to compartmentalize DNA transactions within the mammalian cell nucleus [1], [2].

## Materials and Methods

### Constructs and construction of stable cell lines

pBABE-Puro GFP-lamin A was a gift of Tom Misteli (Addgene plasmid 17662). dsRed-53BP1 mTudor domain, consisting of amino acids 1220-1714 of 53BP1 in the vector pDsRed-monomer-N1 was a kind gift of Nabieh Ayoub and used for expression in human U2OS and 293T cells (from the American Type Culture Collection). For expression in murine embryonic fibroblasts [30], dsRed-53BP1TD was subcloned into pBABE-Hygro (gift of Bob Weinberg, Addgene plasmid 1765) by a two-step cloning procedure. dsRed-53BP1TD was amplified by PCR using the following primers, which introduced 5′ and 3′ BamH1 and SnaB1 sites respectively: 5′ATGGATCCGCTACCGGTCGCCAC CAT3′, 5′ATTACGTAATCCTTATTCACCGGTGTT3′. This PCR product was first blunt end cloned into the vector pCR-blunt (Invitrogen) and then cut out using BamH1 and SnaB1, and ligated into pBABE-Hygro, also cut with the same enzymes. U2OS stable cell lines were produced by selection in 0.6 µg/ml puromycin and/or 1 mg/ml neomycin after seeding at single cell density to obtain a clonal population. They were transfected in Lipofectamine 2000 (Invitrogen) according to the manufacturer's instructions. Murine embryonic fibroblast stable cell lines were produced by retroviral transduction using virus packaged from Ampho Phoenix 4 cells (Orbigen), followed by selection in 125 µg/ml hygromycin and 125 µg/ml puromycin.

### Chemicals and reagents

Etoposide was from Enzo Life Sciences and used at a dose of 5 µM for 30 min unless otherwise stated. Adriamycin was from Sigma and was used at 5 µM for one hour.

### Live cell time-lapse microscopy, laser-induced DNA damage, FRAP and fluorescence correlation spectroscopy

Cells were imaged in L15 CO_2_ independent media (Gibco) at 37°C, in No.1 glass bottom culture dishes (MatTek) on a Leica SP5 confocal microscope with a 40× NA 1.4 lens and Leica application suite software. DNA damage induction in live cells was either focally in a circle of diameter ∼1 µm using a 405 nm diode laser operating at 100% for 1.2 s after pre-sensitization with 10 µg/ml Hoechst 33342, or directly with an Andor micropoint 365 nm pulsed laser with an average power of 750 µW operating at 50% attenuation. PMT detector gain and offset was adjusted so that pixels were not saturated or clipped off, keeping imaging parameters constant between samples. Maximum intensity z-projections when used were formed in ImageJ, and brightness and contrast was altered for display purposes but always consistently across all samples and not before quantitation. GFP-lamin A and dsRed-53BP1TD dynamic behaviour was followed by timelapse imaging using 488/561 nm lasers taking 512×512 pixel images at one minute intervals, with a z-slice depth of ∼1 µm. FRAP photobleaching was performed using 100% of a 488 nm argon laser for 1.2 s in a circular region of size 1 µm in diameter and imaging thereafter was performed using a 488 nm laser taking 256×256 pixel images at scan speed 700 Hz, producing a picture every 0.4 s. Images were analysed using Leica LAS AF software to draw regions of interest and export fluorescence intensity data into Microsoft Excel. Corrections for imaging background and bleaching were made in Excel and graphs were plotted using GraphPad Prism. Curves were fitted in GraphPad Prism using the one phase association equation 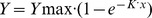 where 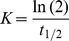 to yield t_1/2_ and plateau (*Y*_max_) values. FCS measurements were taken as described previously [37].

### *In situ* proximity ligation assay (PLA)

*In situ* proximity ligation assay (PLA) was performed with a Duolink kit (Olink Bioscience, Uppsala, Sweden) according to the manufacturer's instructions, with some minor modifications. The format was 96-well (Nunc glass bottomed), using 40 µl reactions. During incubations, plates were sealed with Parafilm and semi-immersed in pre-warmed water in order to ensure optimal staining reproducibility. Antibodies to be used in PLA were first carefully optimised using standard immunofluorescence, to determine the optimal dilution which gave specific staining. The specificity of the assay was confirmed using both positive and negative control readings; positive controls consisted of incubations with two different antibodies targeting GFP and lamin A respectively in GFP-lamin A expressing cells, or using two different lamin A/C antibodies. Negative controls consisted of single antibody incubations, which usually gave some background level. The normalised PLA signal (PLA^n^) was defined as: 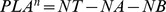, where NT  =  the total number of PLA dots with double antibody incubation, and NA or NB  =  the total number of dots in the nucleus with each single antibody incubation. PLA signal was quantified using a Cellomics Arrayscan, whereby cell nuclei were identified based on Hoechst 33342 staining and PLA dots were counted inside these regions using the “Target activation” Bioapplication.

### Antibodies, western blotting and immuno-pulldown

Western blots were performed by standard protocols: cells were lysed in RIPA buffer (50 mM TrisHCl pH 8, 150 mM NaCl, 1% NP40, 0.5 M Sodium Deoxycholate, 0.1% SDS, 25 mM NaF, 1 mM Na_3_VO_4_, complete protease inhibitor cocktail (Roche)) for 20 min on ice and protein concentration was quantitated using the bicinchoninic acid method to load 50–75 ug of protein. Antibodies used were: anti-dsRed 1∶1000 (Clontech, 632496), anti-GFP 1∶1000 (Clontech JL-8, 632380), anti-lamin A/C 1∶1000 (H110 sc-20681), anti-H2AX 1∶1000 (Sigma 3F4, WH0003014M5; Abcam ab11175), anti-53BP1 1∶2000 (Novus biologicals). Immuno-pulldown was performed with a polyclonal anti-GFP antibody (Clontech living colours full-length A.v. polyclonal antibody (632459)) overnight at 4°C using 1 µl per 1 mg of protein lysate and protein A beads. Lysate was produced using NP40 buffer (50 mM Hepes pH 7.4, 100 mM NaCl, 0.5% NP-40, 1 mM EDTA, 20 mM beta-glycerophosphate, 1 mM sodium orthovanadate, 1 mM DTT, complete protease inhibitor cocktail (Roche), PhosSTOP (Roche) and Benzonase (Novagen) 0.25 U/µl). Immune complexes were washed with NP40 buffer and eluted with sample buffer for analysis by 4–12% Bis-Tris gel (Invitrogen).

### Immunofluorescence

Cells were fixed in 4% paraformaldehyde (Agar Scientific) followed by blocking and antibody incubations in 3% bovine serum albumen (Fischer Scientific). Antibodies used: anti-lamin A/C (Abcam 26300 1∶500; Abcam 133A2 1∶2000), anti-γH2AX Ser139 1∶2000 (JBW301 Millipore), anti-H2AX 1∶2000 (Abcam ab11175).

### DNA repair foci tracking in live cells

DNA repair foci tracking in live cells was performed essentially as described by [14]. Time series were analysed in ImageJ using the Stackreg translation plugin [38] and foci were tracked either through manual identification in each frame using Pointpicker plugin. Six foci were tracked in each time series and the x and y coordinates of the trajectories were analysed in Excel software, correcting for cell mobility using the equations described by [14] to obtain the cumulative distance travelled.

## Supporting Information

Figure S1 Validation of dsRed-53BP1TD as a marker of sites of DNA damage. (**A**) Cells were grown on coverslips, fixed with 4% formaldehyde and stained with anti-γH2AX antibody. dsRed-53BP1TD dots co-localise with γH2AX foci. (**B**) Living dsRed-53BP1TD cells were imaged with confocal fluorescence microscopy (-IR), then irradiated with 10Gy IR, and imaged again using the same imaging settings. There is an increase in the dsRed-53BP1TD signal after DNA damage.
(PDF)

Figure S2 Control PLA measurements. (**A**–**D**) PLA control samples in GFP-lamin A cells stained with; (**A**) anti-lamin A/C and anti-GFP, both targeting stably expressed GFP-lamin, (**B**) anti-lamin A/C only (**C**) anti-GFP only. (**D**) Positive control PLA sample in human U2OS cells using two different antibodies targeting lamin A/C. PLA signal (red dots) co-localises with GFP-lamin A signal, indicating its specificity, and is proportional to expression level. (**E**) Negative control PLA reactions in H2AX−/− and H2AX+/+ murine embryonic fibroblasts after IR showing the mean +/− s.e.m number of PLA dots in >1000 cells.
(PDF)

Figure S3 Expression of GFP-lamin A and dsRed-53BP1TD in LMNA−/− murine embryonic fibroblasts. (**A**) The histogram shows the average fluorescence intensity of GFP-lamin A in the nucleus of 1000 cells measured by semi-automated fluorescence microscopy, indicating efficient expression of GFP-lamin A. (**B**) Histogram of dsRed-53BP1TD nuclear fluorescence in 12000 matched LMNA−/− and LMNA−/−, GFP-lamin A stably transfected murine embryonic fibroblasts, showing the same expression levels in different genotypic backgrounds.
(PDF)

Video S1 Live cell time-lapse imaging of stably expressed GFP-lamin A (green) and dsRed-53BP1TD (red) during DNA damage induction. DNA damage was induced with a 405 nm laser in U2OS cells pre-sensitized using Hoechst 44432. Z-stack images were collected using a laser scanning confocal microscope (Leica SP5) at one minute intervals and later formed into maximum intensity z-projections using ImageJ software. This video displays the RED (dsRed53BP1TD) channel alone.
(WMV)

Video S2 As in Video S1, except that this video displays the GREEN (GFP-Lamin A) channel alone.
(WMV)

Video S3 As in Videos S1 and S2, except that this video displays the merged red and green channels.
(AVI)

Video S4 Live cell time-lapse imaging of stably expressed GFP-lamin A (green) and dsRed-53BP1TD (red) during DNA damage repair. DNA damage was induced with a 365 nm laser in U2OS cells and z-stack images were collected using a laser scanning confocal microscope (Leica SP5) at one minute intervals for 160 minutes, starting at 60 minutes post DNA damage. Maximum intensity z-projections were formed using ImageJ software.
(AVI)
