# Supplementary figures and images for: A-Type Lamins Maintain the Positional Stability of DNA Damage Repair Foci in Mammalian Nuclei
Source: PLoS One. 2013 May 2;8(5):e61893. doi: 10.1371/journal.pone.0061893 (PMC3642183; doi:10.1371/journal.pone.0061893)

Figure S1

A

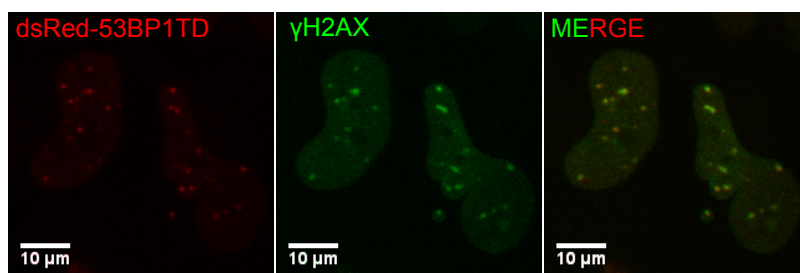

B

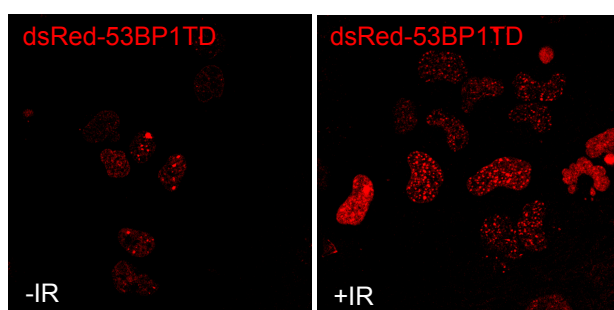

Supplement: Figure S1 — Validation of dsRed-53BP1TD as a marker of sites of DNA damage. (A) Cells were grown on coverslips, fixed with 4% formaldehyde and stained with anti-γH2AX antibody. dsRed-53BP1TD dots co-localise with γH2AX foci. (B) Living dsRed-53BP1TD cells were imaged with confocal fluorescence microscopy (-IR), then irradiated with 10Gy IR, and imaged again using the same imaging settings. There is an increase in the dsRed-53BP1TD signal after DNA damage. (PDF) [file pone.0061893.s001.pdf]

# Figure S2

GFP-lamin A DAPI (DNA) PLA (Lamin-GFP interaction)

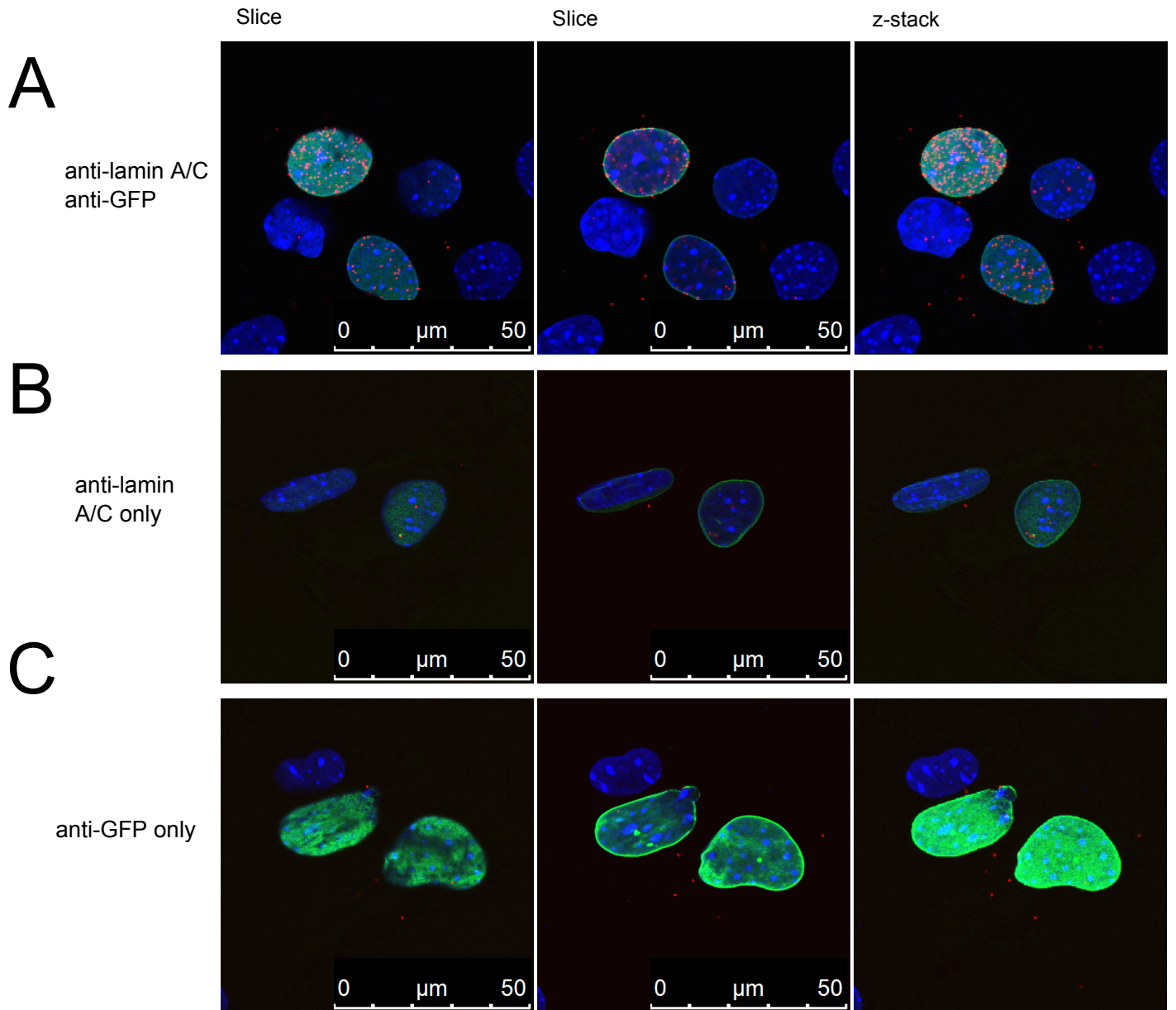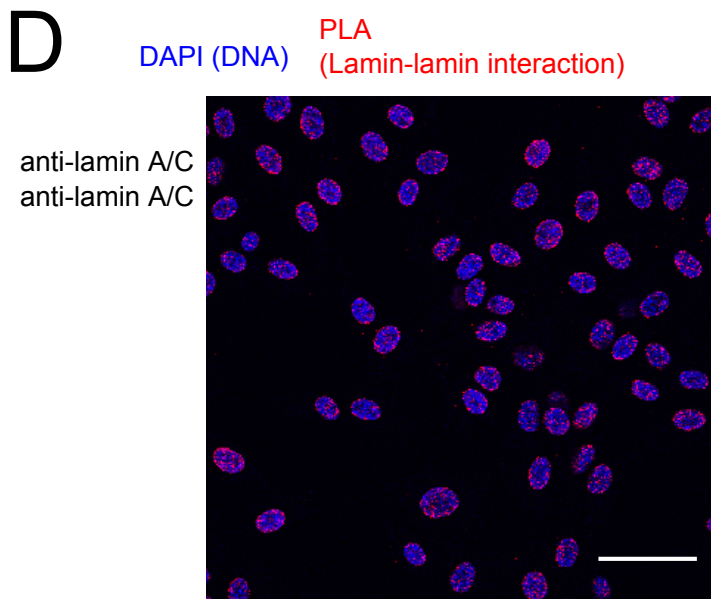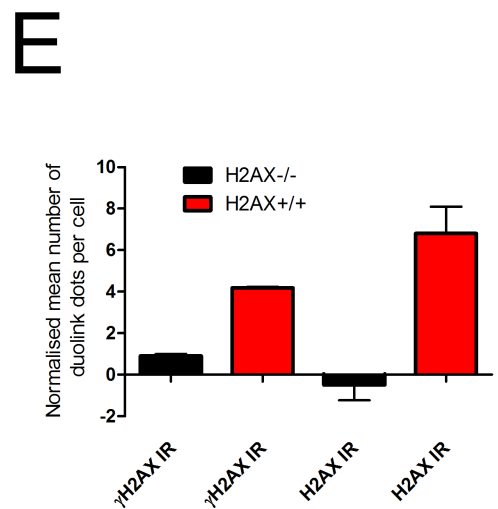

Supplement: Figure S2 — Control PLA measurements. (A–D) PLA control samples in GFP-lamin A cells stained with; (A) anti-lamin A/C and anti-GFP, both targeting stably expressed GFP-lamin, (B) anti-lamin A/C only (C) anti-GFP only. (D) Positive control PLA sample in human U2OS cells using two different antibodies targeting lamin A/C. PLA signal (red dots) co-localises with GFP-lamin A signal, indicating its specificity, and is proportional to expression level. (E) Negative control PLA reactions in H2AX−/− and H2AX+/+ murine embryonic fibroblasts after IR showing the mean +/− s.e.m number of PLA dots in >1000 cells. (PDF) [file pone.0061893.s002.pdf]

# Figure S3

## A

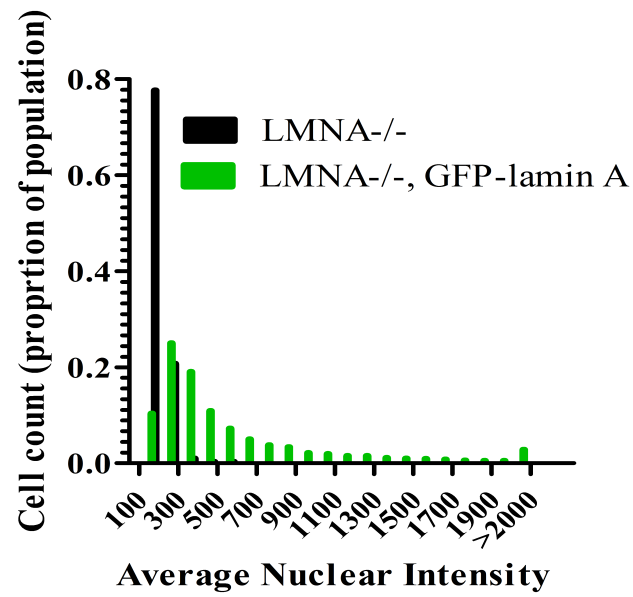

## B

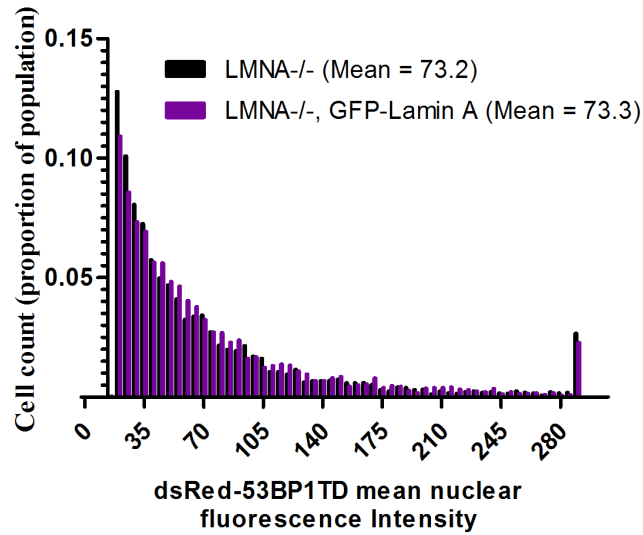

Supplement: Figure S3 — Expression of GFP-lamin A and dsRed-53BP1TD in LMNA−/− murine embryonic fibroblasts. (A) The histogram shows the average fluorescence intensity of GFP-lamin A in the nucleus of 1000 cells measured by semi-automated fluorescence microscopy, indicating efficient expression of GFP-lamin A. (B) Histogram of dsRed-53BP1TD nuclear fluorescence in 12000 matched LMNA−/− and LMNA−/−, GFP-lamin A stably transfected murine embryonic fibroblasts, showing the same expression levels in different genotypic backgrounds. (PDF) [file pone.0061893.s003.pdf]
